# The taxonomy of the model filamentous fungus *Podospora anserina*

**DOI:** 10.3897/mycokeys.75.55968

**Published:** 2020-11-25

**Authors:** S. Lorena Ament-Velásquez, Hanna Johannesson, Tatiana Giraud, Robert Debuchy, Sven J. Saupe, Alfons J.M. Debets, Eric Bastiaans, Fabienne Malagnac, Pierre Grognet, Leonardo Peraza-Reyes, Pierre Gladieux, Åsa Kruys, Philippe Silar, Sabine M. Huhndorf, Andrew N. Miller, Aaron A. Vogan

**Affiliations:** 1 Systematic Biology, Department of Organismal Biology, Uppsala University, Norbyvägen 18D, 752 36 Uppsala, Sweden Uppsala Univeristy Uppsala Sweden; 2 Ecologie Systématique Evolution, CNRS, Université Paris-Saclay, AgroParisTech, 91400, Orsay, France Université Paris-Saclay Orsay France; 3 Université Paris-Saclay, CEA, CNRS, Institute for Integrative Biology of the Cell (I2BC), 91198, Gif-sur-Yvette, France Université Paris-Saclay Gif-sur-Yvette France; 4 IBGC, UMR 5095, CNRS Université de Bordeaux, 1 rue Camille Saint Saëns, 33077, Bordeaux, France Université de Bordeaux Bordeaux France; 5 Laboratory of Genetics, Wageningen University, Arboretumlaan 4, 6703 BD, Wageningen, Netherlands Wageningen University Wageningen Netherlands; 6 Instituto de Fisiología Celular, Departamento de Bioquímica y Biología Estructural, Universidad Nacional Autónoma de México, Mexico City, Mexico Universidad Nacional Autónoma de México Mexico City Mexico; 7 UMR BGPI, Université de Montpellier, INRAE, CIRAD, Institut Agro, F-34398, Montpellier, France Université de Montpellier Montpellier France; 8 Museum of Evolution, Botany, Uppsala University, Norbyvägen 18, 752 36, Uppsala, Sweden Uppsala University Uppsala Sweden; 9 Université de Paris, Laboratoire Interdisciplinaire des Energies de Demain (LIED), F-75006, Paris, France Université de Paris Paris France; 10 Botany Department, The Field Museum, Chicago, Illinois 60605, USA The Field Museum Chicago United States of America; 11 Illinois Natural History Survey, University of Illinois, Champaign, IL 61820, USA University of Illinois Champaign United States of America

**Keywords:** phylogenetics, Podospora, Podosporaceae, taxonomy

## Abstract

The filamentous fungus *Podospora anserina* has been used as a model organism for more than 100 years and has proved to be an invaluable resource in numerous areas of research. Throughout this period, *P. anserina* has been embroiled in a number of taxonomic controversies regarding the proper name under which it should be called. The most recent taxonomic treatment proposed to change the name of this important species to *Triangularia anserina*. The results of past name changes of this species indicate that the broader research community is unlikely to accept this change, which will lead to nomenclatural instability and confusion in literature. Here, we review the phylogeny of the species closely related to *P. anserina* and provide evidence that currently available marker information is insufficient to resolve the relationships amongst many of the lineages. We argue that it is not only premature to propose a new name for *P. anserina* based on current data, but also that every effort should be made to retain *P. anserina* as the current name to ensure stability and to minimise confusion in scientific literature. Therefore, we synonymise *Triangularia* with *Podospora* and suggest that either the type species of *Podospora* be moved to *P. anserina* from *P. fimiseda* or that all species within the Podosporaceae be placed in the genus *Podospora*.

## Introduction

*Podospora anserina* is a model filamentous fungus that has been at the forefront of molecular biology and genetics for over 100 years (Silar 2020). It has been instrumental in numerous important biological breakthroughs, such as the discovery of eukaryotic plasmids and prions and has been monumental in furthering the fields surrounding aging/senescence, meiotic drive, allorecognition (known as heterokaryon incompatibility in fungi), sexual reproduction and genome defence (Esser 1974; Saupe et al. 2000; Saupe 2007; Silar 2013, 2020; Grognet et al. 2019; Vogan et al. 2019). Along with its role in basic research, *P. anserina* has also caught the attention of industry, where it is used as a source of enzymes that play various roles in the degradation of plant material (Couturier et al. 2016). More recently, *P. anserina* has burst into the genomics era with one of the first published fungal genomes (Fitzpatrick et al. 2006), released in 2008 (Espagne et al. 2008). In the last year, 10 additional chromosome level assemblies of *P. anserina* have been released in concert with the genome of the closely-related species, *P. comata* and *P. pauciseta* (Silar et al. 2019; Vogan et al. 2019). Future projects will expand on this role even further. Wageningen University hosts a collection of strains isolated from the same locale, spanning 30 years of sampling (van der Gaag et al. 1998, 2000; Vogan et al. 2020), which have now all been sequenced and chromosome level assemblies (in preparation) have been produced for the remaining four species of the *Podospora anserina* species complex (Boucher et al. 2017). Therefore, the role of *P. anserina* will continue to be central to many fields in biology and, indeed, likely see use in new fields as new data become public.

The taxonomic history of *Podospora anserina* has been a long and complex one (reviewed in Silar 2020). *Podospora* is a member of the Sordariales and has been traditionally grouped within the family Lasiosphaeriaceae, which itself is an artificial assemblage of genera whose main diagnostic is that they do not belong to the Sordariaceae (Lundqvist 1972). Species within the Lasiosphaeriaceae were divided into genera, based primarily on ascospore morphology, but molecular phylogenies have revealed that these characters do not represent synapomorphies and that most of the genera are polyphyletic (Huhndorf et al. 2004; Miller and Huhndorf 2005). A broad phylogenetic treatment of coprophilous Lasiosphaeriaceae defined four separate clades, with species of *Podospora* represented in all clades, exemplifying the lack of informative morphology amongst these fungi (Kruys et al. 2015). Moreover, the taxon *P. anserina* itself has survived multiple attempts to rename it in the past, which were unsuccessful in part due to how deeply ingrained the name is in the genetics and molecular biology research community (Boucher et al. 2017; Silar 2020). Recently, this taxonomic mess was stumbled upon by researchers attempting to clean up the distantly-related genus *Thielavia* (Wang et al. 2019). The authors defined the clade containing the type species of *Podospora* (*P. fimiseda*, incorrectly referred to as *P. fimicola* in Wang et al. 2019) as the Podosporaceae (formerly Lasiosphaeriaceae IV) based on a four-marker phylogeny and further divided this clade into three genera: *Podospora*, *Triangularia* and *Cladorrhinum*. As their analyses suggested that *P. anserina* is more closely related to the type species of *Triangularia* (*T. bambusae*), they proposed the new combination, *Triangularia anserina* (Wang et al. 2019).

It is the opinion of the authors here that the taxonomic change of *P. anserina* is both premature and against the ideals of the International Code of Nomenclature for algae, fungi and plants (ICN), as stated in the preamble (Turland et al. 2018). Foremost, it is unlikely that *T. anserina* will be adopted by many of the researchers that rely on it as a model organism, leading to instability of the name. Furthermore, the phylogeny upon which the change was based has a sparse sampling of the diversity of the family and used only four markers. Here, we demonstrate that there is a lack of information amongst the markers currently sequenced in this group and argue that more data are needed before formal taxonomic changes are made for *Podospora*. Ultimately, the best solution for taxonomic stability in the Podosporaceae will be to change the type species of *Podospora* from *P. fimiseda*, which was conserved in 1972 (Nicolson et al. 1984), to *P. anserina* and to only define new genera once more sequence data are available, likely in the form of whole genome sequences.

## Methods

### Sequences and strains

We generated sequences of 29 strains from 27 species in the Podosporaceae family for markers typically used in molecular phylogenetic studies of Sordariomycetes (including Wang et al. 2019): the ribosomal large subunit (LSU), beta-tubulin (Btub) and RNA polymerase II (rpb2) (Table 1). Sequences were generated as per Huhndorf et al. 2004 and Miller and Hundorf 2005. In brief, DNA was extracted from dried ascomata or multispore isolates of growing cultures using a DNeasy Mini Plant extraction kit (Qiagen Inc., Valencia, California), following manufacturer’s recommendations with the exception that tissues were ground in 100 ml AP1 buffer rather than liquid nitrogen. Markers were amplified with the primers listed in Suppl. material 4: Table S1 and sequenced on an Applied Biosystems 3100 automated DNA sequencer. Sequences were deposited in GenBank with accession numbers MT731502–MT731583. In addition, we collected available sequences from GenBank and the NBRC culture collection for all strains suspected to fall within the Podosporaceae for the above markers, as well as the fungal barcode ITS (Schoch et al. 2012). For Btub, two regions are often used in phylogenetic analyses. We sequenced the C-terminal domain of Btub with only a single intron (Btub2), but included sequences from databases that correspond to the upstream intron-rich GTPase domain (Btub1) to maximise the number of taxa. When available sequences of markers overlapped with ones that were generated for this study exactly, but had longer flanks, those sequences were merged (two GenBank codes in Table 1). Finally, we included representative strains of the type species of the other families within the Sordariales, as well as the type species of *Zopfiella* and *Cercophora*, which have many representatives within the Podosporaceae, as outgroups. The alignment of all concatenated markers is deposited in TreeBase (http://purl.org/phylo/treebase/phylows/study/TB2:S26777).

**Table 1.** Strains and markers included in this study. Sequences generated in this study are in bold.

Strain	Species	Clade	ITS	LSU	BTub1	BTub2	RPB2
CBS 539.89**T**	Apiosordaria backusii	A	MK926866	**MT731508**	MK926966	**MT731549**	**MT731570**
CBS 106.77	Apiosordaria backusii	A	MK926867	AY780051	MK926967	AY780085	AY780149
CBS 304.81**T**	Apiosordaria effusa	A	3086201^b^	3086201^b^			
CBS 390.84**T**	Apiosordaria longicaudata	A	954801^b^	**MT731505**		**MT731544**	**MT731580**
CBS 244.71**T**	Apiosordaria stercoraria	A	MH860096	968201^b^			
CBS 629.82**T**	Apiosordaria tenuilacunata	A	MH861532	**MT731507**		**MT731548**	**MT731569**
CBS 363.84**T**	Apiosordaria tetraspora	A		**MT731506**		**MT731545**	**MT731581**
NBRC 30422	Apiosordaria verruculosa ^a^	A	3042201^b^	3042201^b^			
NBRC 30423	Apiosordaria verruculosa ^a^	A	3042301^b^	3042301^b^			
CBS 148.77	Apiosordaria verruculosa ^a^	A	MK926874	**MT731510**	MK926974	**MT731546**	**MT731577**
F-152365	Apiosordaria verruculosa ^a^	A		AY346258		AY780086	AY780150
CBS 550.66	Apiosordaria verruculosa ^a^	A		**MT731511**		**MT731547**	**MT731579**
CBS 432.64	Apiosordaria verruculosa ^a^	A	MH858479	MH870111			
CBS 433.64	Apiosordaria verruculosa ^a^	A	MH858480	MH870112			
CBS 268.67	Apiosordaria verruculosa ^a^	A	MH858965				
NBRC 31170**T**	Apiosordaria yaeyamensis	A	LC146720	LC146720			
CBS 120.289	Arnium arizonense	A	KU955584	KF557671		**MT731535**	**MT731563**
S 18211-c	Arnium arizonense	A		KF557668		KF557706	
UPS 724	Arnium arizonense	A		KF557669		KF557707	
E00204509	Arnium arizonense	A		KF557670		KF557708	
CBS 307.81**T**	Cercophora samala	A	MH861345	MH873104			
CBS 109.93	Cercophora samala	A	AY999134	AY999111	AY999140		
CBS 125293**T**	Cercophora squamulosa	A	MH863506				
JF 06314**T**	Cercophora aquatica	A	JN673038	JN673038			
SMH 3431	Cercophora striata	A		AY780065		AY780108	AY780169
SMH 4036	Cercophora striata	A	KX348038	AY780066			
CBS 290.75**T**	Cladorrhinum microsclerotigenum	A	FN662475	FN662476			
CBS 301.90**T**	Cladorrhinum phialophoroides	A	FM955444	FR692344	KT291718/MK926971		MK876833
S**T**	Podospora anserina	A	Genomic	Genomic	Genomic	Genomic	Genomic
CBS 455.64	Podospora anserina	A		**MT731521**		**MT731540**	**MT731564**
CBS 533.73	Podospora austroamericana	A		**MT731509**		**MT731539**	**MT731582**
CBS 724.68**T**	Podospora austroamericana	A	MK926865	AY999101	MK926965		MK876827
CBS 405.72	Podospora platensis	A	MH860505	**MT731514**		**MT731550**	**MT731571**
CBS 251.71**T**	Podospora praecox	A	MH860101	MH871877			
FMR 12787	Podospora setosa	A		KP981441		KP981569	KP981624
CBS 435.50	Podospora setosa	A	GQ922533	MH868219			
CBS 311.58	Podospora setosa	A	MK926872	MK926872	MK926972		MK876834
CBS 369.59	Podospora setosa	A	MK926873	**MT731515**	MK926973	**MT731551**	**MT731572**
CBS 265.70	Podospora tarvisina	A	MH859600	**MT731516**		**MT731552**	**MT731573**
CBS 313.58**T**	Podospora unicaudata	A	MH857799	**MT731513**		**MT731554**	**MT731575**
CBS 240.71	Podospora unicaudata	A	MH860093	MH871871			
CBS 165.74	Triangularia angulispora	A		**MT731517**		**MT731543**	**MT731568**
NBRC 30009	Triangularia bambusae ^a^	A	3000901^b^	3000901^b^			
CBS 352.33**T**	Triangularia bambusae ^a^	A	MK926868	**MT731518**	MK926968	**MT731541**	**MT731578**/MK876830
CBS 381.68**T**	Triangularia batistae	A	MH859162	**MT731519**		**MT731542**	**MT731576**
IFO 30296	Zopfiella longicaudata	A	AY999131	AY999109			
FMR 12365	Zopfiella longicaudata	A		KP981448		KP981574	KP981631
FMR 12782	Zopfiella longicaudata	A		KP981449		KP981575	KP981632
CBS 252.57**T**	Zopfiella longicaudata	A	MK926869	**MT731503**	MK926969	**MT731536**	**MT731567**
CBS 256.71	Zopfiella longicaudata	A	MH860106	MH871881			
CBS 257.78	Zopfiella longicaudata	A		**MT731504**		**MT731537**	**MT731565**
CBS 971.73	Zopfiella longicaudata	A		**MT731502**		**MT731538**	**MT731566**
CBS 671.82**T**	Zopfiella ovina	A	MH861539	**MT731512**		**MT731553**	**MT731574**
CBS 127120	Zopfiella sp.	A	MH864427	MH875865			
IFO 32904	Zopfiella tetraspora	A	AY999130	AY999108	AY999139		
CBS 245.71	Zopfiella tetraspora	A	MH860097	**MT731520**			**MT731583**
CBS 120012	Arnium olerum ^a^	B		**MT731522**		KF557718	**MT731561**
SMH3253	Arnium olerum ^a^	B		KF557690			
FMR 13412	Arnium sp.	B		KP981428		KP981555	KP981610
S	Arnium tomentosum	B		KF557691		KF557720	
SMH 4089	Cercophora coprophila	B		KF557692			
IFO 32091	Cercophora coprophila	B	AY999136	AY999112	AY999141		
SMH 3794	Cercophora coprophila	B		AY780058		AY780102	AY780162
CBS 120013**T**	Cercophora grandiuscula	B	GQ922544	**MT731524**		**MT731530**	**MT731562**
ATCC 200395	Cercophora terricola	B		AY780067		AY780109	AY780170
CBS 180.66**T**	Cladorrhinum foecundissimum ^a^	B	MK926856	FR692343	KT291717/MK926956		MK876818
CBS 182.66	Cladorrhinum foecundissimum ^a^	B	MH858768				
BCCM 6980	Cladorrhinum foecundissimum ^a^	B	KT321080	KT312993	KT291721		
CGMCC3.17921	Cladorrhinum globisporum	B					KY883234
LC5415	Cladorrhinum globisporum	B	KU746680	KU746726	KU746771		
TTI-313	Podospora australis	B	KX015765	KX015765			
LyRS93415	Podospora australis	B		KF557696			
LyRS92471	Podospora australis	B		KF557695			
CBS 322.70**T**	Thielavia hyalocarpa	B	MK926857	MK926857	MK926957		MK876819
CBS 102198	Thielavia hyalocarpa	B	MK926858	MK926858	MK926958		MK876820
CBS 433.96**T**	Thielavia intermedia	B	MK926859	MK926859	MK926959		MK876821
CBS 100257	Thielavia intermedia	B	MK926860	MK926860	MK926960		MK876822
CBS 389.84	Zopfiella leucotricha	B	982801^b^	**MT731523**			**MT731560**
CBS 463.61	Zopfiella leucotricha	B	MH858107	MH869684			
CGMCC 3.15230	Apiosordaria hamata	C	KP878306	KP878304			
NBRC 30406	Apiosordaria jamaicensis	C	3040601^b^	3040601^b^			
CBS 672.70**T**	Apiosordaria jamaicensis	C	MH859895	**MT731527**		**MT731534**	**MT731556**
FMR 6363	Apiosordaria nigeriensis	C	AJ458184				
CBS 713.70**T**	Apiosordaria sacchari	C	MH859915	KP981425		KP981552	KP981607
CBS 259.71**T**	Apiosordaria spinosa	C		MH877809			
CBS 154.77	Apiosordaria striatispora	C	MH861043	**MT731529**			**MT731559**
CBS 258.71**T**	Apiosordaria tuberculata	C	MH860107	MH871882			
SMH 4021	Cercophora costaricensis	C		AY780059		AY780103	AY780163
SMH 3200	Cercophora sp.	C		AY780055		AY780098	AY780159
INTA-AR 70**T**	Cladorrhinum australe	C	KT321062	KT312976	KT291700		
CBS 304.90**T**	Cladorrhinum bulbillosum	C	MK926861	MK926861	MK926961		MK876823
CBS 126090**T**	Cladorrhinum flexuosum	C	MH864075	FN662477			
CBS 303.90	Cladorrhinum samala	C	FM955447	FR692338			
CBS 302.90	Cladorrhinum samala	C		KT312992	KT291719		
NBRC 107619	Cladorrhinum sp.	C	12744402^b^	12744401^b^			
CBS 482.64**T**	Podospora fimiseda ^a^	C	MK926862	**MT731525**	MK926962	**MT731531**	**MT731557**
CBS 990.96	Podospora fimiseda ^a^	C	AY515361	AY346296	MK926963	AY780133	AY780190
CBS 257.71	Zopfiella inermis	C		**MT731526**		**MT731533**	**MT731555**
CBS 286.86**T**	Zopfiella macrospora	C	MH861958	**MT731528**		**MT731532**	**MT731558**
CBS 643.75A**T**	Cladorrhinum brunnescens		FM955446	FR692346			
**Outgroups**							
CBS 148.51	Chaetomium globosum ^a^	Out	Genomic	Genomic	Genomic	Genomic	Genomic
CBS 160.62	Chaetomium globosum ^a^	Out	KT214565	KT214596	KT214742		KT214666
FMR 13414	Diplogelasinospora princeps ^a^	Out		KP981431		KP981559	KP981614
SMH 1538	Lasiosphaeria ovina ^a^	Out		AF064643		AF466046	AY600287
SMH 4106	Sordaria fimicola ^a^	Out		AY780079		AY780138	AY780194
CBS 230.78	Zopfiella tabulata ^a^	Out	MK926854	MK926854	MK926954		MK876816
CBS 120402	Cercophora mirabilis ^a^	Out		KP981429		KP981556	KP981611

T Strain is the Type of the species; ^a^ Type species of genus; ^b^ Sequences taken from NBRC

### Phylogenetic analyses

Each locus was aligned using the online server of MAFFT v. 7.467 (https://mafft.cbrc.jp/alignment/server/; (Katoh et al. 2019) with default settings, followed by manual curation adjusting for the coding frame of the protein-coding markers. We concatenated all alignments and performed a Maximum Likelihood analysis using IQ-TREE v 1.6.8 (Nguyen et al. 2015; Kalyaanamoorthy et al. 2017) with an extended model selection (-m MFP) and 100 standard bootstrap pseudo-replicates. In addition, each individual marker and combinations of markers were analysed as above, but only including sequences that were at least 45% as long as the locus alignment and/or larger than 250 bp. Only strains that consistently showed membership to the Podosporaceae are presented here. The isolates *Podospora brasiliensis* CBS 892.96, *Podospora inflatula* CBS 412.78 and *P. inflatula* CBS 413.82 likely belong to the family, but were excluded due to inconsistent affinities of markers.

### Evaluating phylogenetic signal

To evaluate the phylogenetic signal in our datasets, we followed the approach of Shen et al. (2017), which quantifies the amount of support of particular sites or entire genes for two alternative topologies with respect to a particular branch (termed T1 and T2). We set T1 as the Maximum Likelihood topology produced with the concatenated alignment of all markers and T2 as a topology inferred in the same way but constrained to maintain the Clades A and C (see Results) as sisters. To determine what topology is the most supported for each site of each marker, we calculated the site-wise log-likelihood score using RAxML v. 8.2.12 (Stamatakis 2014) with the options -*f G -m GTRGAMMA.* The output was processed with the scripts *1_sitewise_analyzer.pl* and *2_genewise_analyzer.pl* (Shen et al. 2017) and additional custom scripts available as a full Snakemake (Köster and Rahmann 2018) pipeline at https://github.com/SLAment/Podosporaceae. As a result, we obtained the gene-wise log-likelihood score of each gene for either T1 or T2 and compared them by calculating their difference in likelihood (ΔGLS) following Shen et al. (2017).

## Results

**Figure 1.**
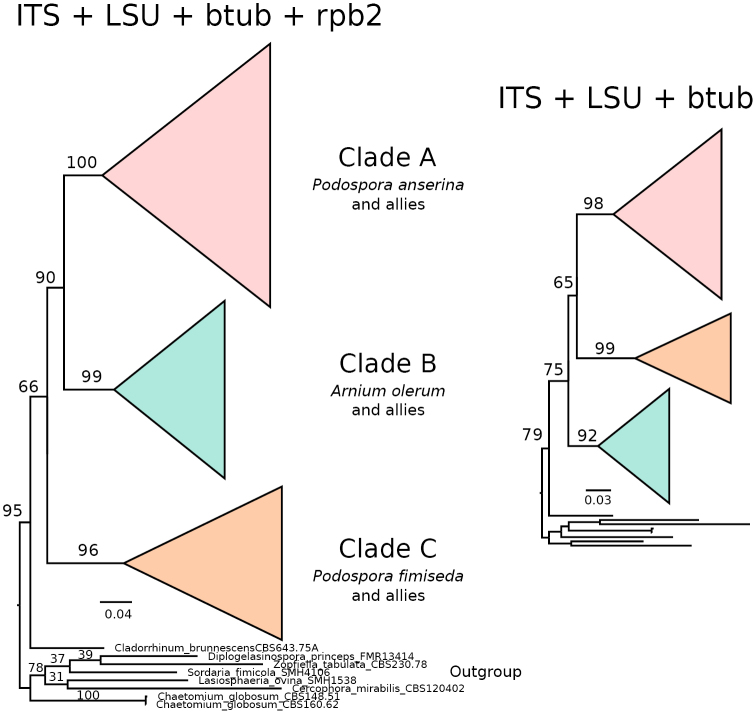
Schematic phylogenetic relationships of the main clades within the Podosporaceae based on Maximum Likelihood analyses of concatenated markers. The three main clades (A, B and C) are strongly supported (bootstrap support values next to relevant branches), but their particular relationship changes depending on the presence of the rpb2 marker. Branches proportional to the scale bar (nucleotide substitutions per site).

**Figure 2.**
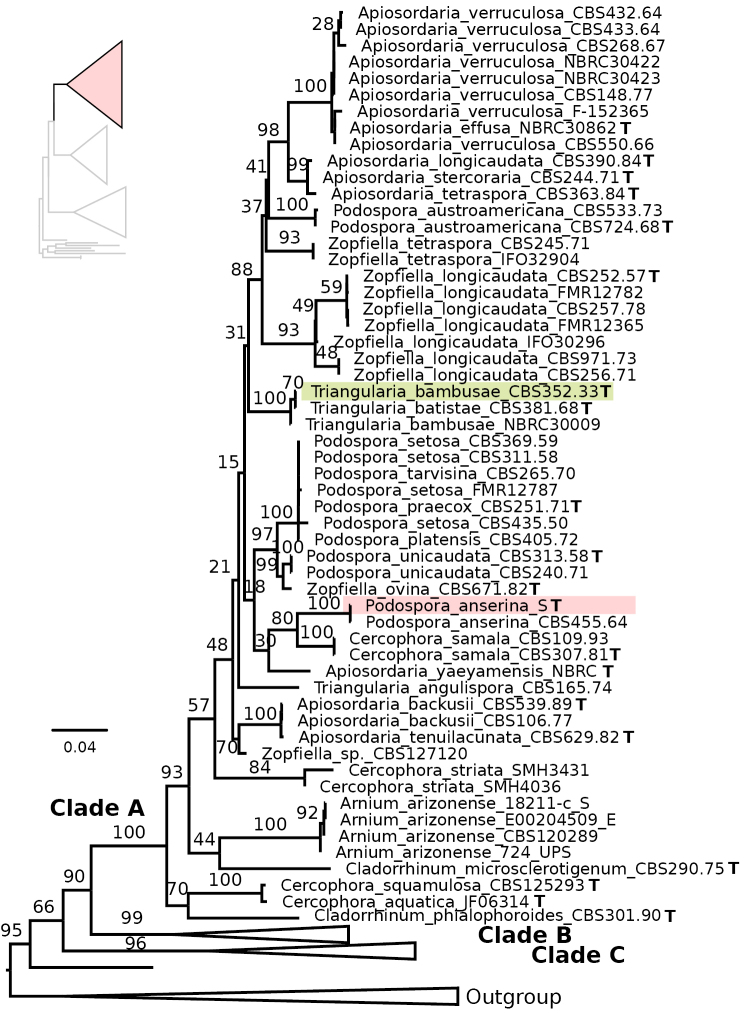
Maximum Likelihood phylogeny of the concatenated analysis of ITS, LSU, Btub and rpb2 for the Podosporaceae, with an emphasis on Clade A. Type strains are indicated with a bold T and those of the focal species *Podospora anserina* and *Triangularia bambusae* are highlighted with coloured boxes. Bootstrap support values are depicted next to their respective branches, but values corresponding to nearly identical sequences are removed for clarity. Branches are proportional to the scale bar (nucleotide substitutions per site).

**Figure 3.**
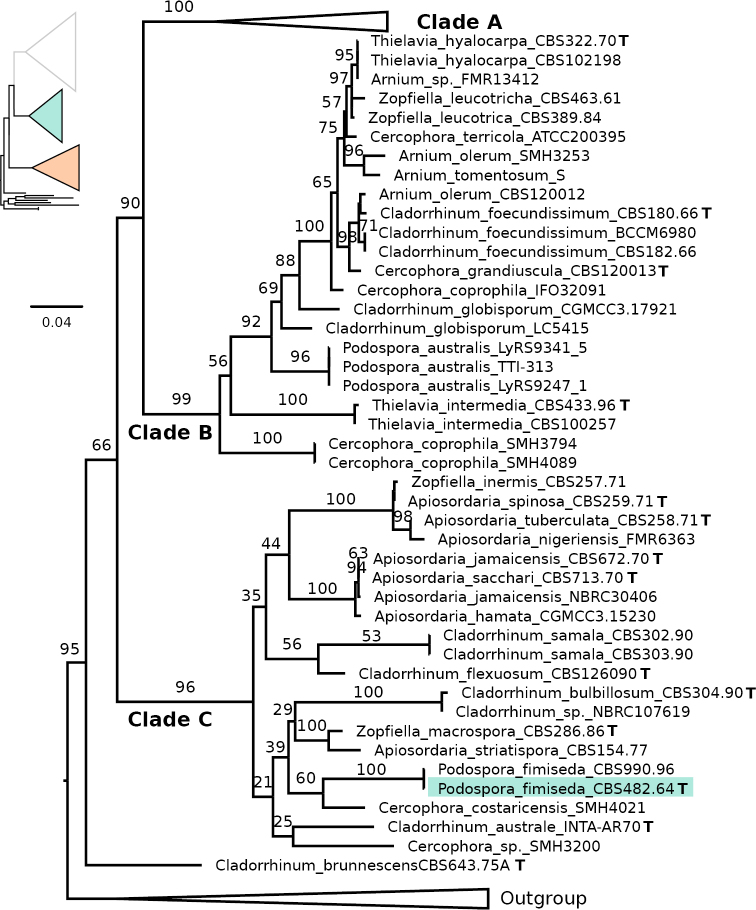
Maximum Likelihood phylogeny of the concatenated analysis of ITS, LSU, Btub and rpb2 for the Podosporaceae, with an emphasis on the clades B and C. Type strains are indicated with a bold T and that of the focal species *Podospora fimiseda* is highlighted with a coloured box. Bootstrap support values are depicted next to their respective branches, but values corresponding to nearly identical sequences are removed for clarity. Branches are proportional to the scale bar (nucleotide substitutions per site).

**Figure 4.**
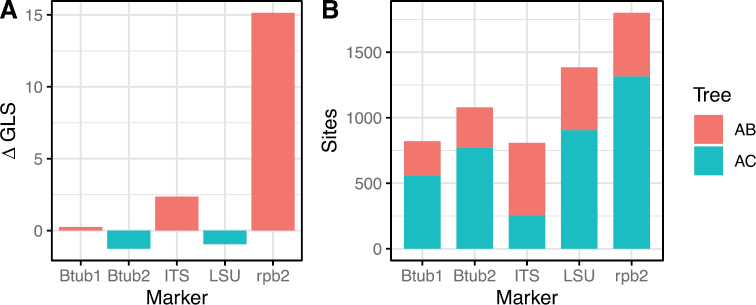
Phylogenetic signal in the available molecular markers for the relationship between clade A and either clade B or C of the Podosporaceae**A** differences in the gene-wise log-likelihood scores (ΔGLS) for each marker, where 0 implies equal support for either of the two alternative sister relationships (A and B or A and C), positive values mean higher support for A and B and negative values higher support for A and C **B** proportion of sites that support each of the two sister relationships within each marker.

Our complete dataset contains 107 taxa and 5895 sites, of which 2110 are variable and 1654 are parsimony informative (Suppl. material 5: Table S2: Supplementary_Table2_Markers). However, combined datasets have a considerable amount of missing data due to the sparse availability of markers for most species. In agreement with previous studies, Maximum Likelihood analyses of all markers reveal that three well-supported clades are resolved within the family, referred to here as Clade A, Clade B and Clade C (Fig. 1; see also Suppl. material 1: Fig. S1 ITSLSU.min0.45–250, Suppl. material 2: Fig. S2 Btub1_and_2.min0.45–250 and Suppl. material 3: Fig. S3 rpb2.min0.45–250). The exception is Btub1 and Btub2, alone or combined, which tend to place members of the outgroup within the ingroup (Suppl. material 2: Fig. S2 Btub1_and_2.min0.45–250). The taxon *Cladorrhinum brunnescens* appears to represent a distinct lineage within the family, but this finding is only based on the rDNA markers, as no other markers are available for this taxon. None of the main clades of the combined dataset shows monophyly for any genera included in the analyses and, for some species, like *Arnium olerum*, representative strains appear to be highly divergent. The focal species of this paper, *P. anserina*, falls within Clade A (Fig. 2), whereas the type species of *Podospora*, *P. fimiseda*, is in Clade C (Fig. 3). While each clade itself is generally well supported for individual markers and combinations of markers, the relationships between the clades are not (Suppl. material 1: Fig. S1 ITSLSU.min0.45–250, Suppl. material 2: Fig. S2 Btub1_and_2.min0.45–250 and Suppl. material 3: Fig. S3 rpb2.min0.45–250). The combined analysis of all markers shows support for the sister relationship of Clades A and B, as reported previously by Wang et al. (2019), but this topology seems to be driven exclusively by the rpb2 marker (Fig. 1). A concatenated analysis of all markers, excluding rpb2, recovers a sister relationship between Clades A and C instead, albeit poorly supported. Except for rpb2, individual markers have generally low ΔGLS values (that is, the difference in likelihood between competing topologies is small), indicating that they have relatively low support for either potential sister relationship (AB or AC). By contrast, rpb2 is strongly biased towards the AB hypothesis (Fig. 4A). Notably, the majority of sites for most markers have higher support for the AC relationship, including rpb2 (Fig. 4B). This suggests that the AC clustering is often favoured by any given site, but only weakly (i.e. the difference in likelihood is very small). Although less frequent, the sites in rpb2 that do support the AB relationship have a large likelihood difference between topologies and likely drive the overall positive ΔGLS value of this gene. Thus, the strong degree of conflict between markers for this internode seems to be driven by a single gene with strong phylogenetic signal (rpb2) and for several other markers without sufficient phylogenetic signal.

## Discussion

With the widespread use of molecular markers to determine the phylogenetic relationships among species, numerous important fungal groups have faced taxonomic challenges including *Cryptococcus* (Hagen et al. 2015; Kwon-Chung et al. 2017), *Zymoseptoria* (Quaedvlieg et al. 2011), *Fusarium* (Geiser et al. 2013), *Magnaporthe* (Zhang et al. 2016) and *Ustilago* (McTaggart et al. 2016; Thines 2016), many of which remain unresolved. Fungi are, of course, not the only group whose phylogenetic and taxonomic concepts are in conflict; the model insect genus, *Drosophila*, is still embroiled in a nomenclatural controversy that has yet to be satisfactorily resolved (O’Grady and Markow 2009; Johnson 2018). In the case presented before us, the issue is, in fact, not so complex as most of these other examples. The taxonomic assignments amongst species and strains of the Sordariales has long been recognised as a difficult problem, with various authors seeking to provide some clarity (Huhndorf et al. 2004; Miller and Huhndorf 2005; Félix 2015; Kruys et al. 2015). It is clear that previous use of morphological characters to designate genera has failed to resolve monophyletic groups, as all genera represented here are not only distributed amongst the three clades within the Podosporaceae, but can also be found in the much more distantly-related clades (Lasiosphaeriacceae I–III *sensu*Kruys et al. (2015)). It is thus apparent that the way forward is to define the genera based on molecular phylogenies.

While previous phylogenetic analyses on the Sordariales in general have been informative, the lack of resolution remains a pervasive issue (e.g. Kruys et al. 2015). Our results suggest that the molecular markers, typically used for the study of this group, have a relatively low phylogenetic signal for a number of key internodes. Within Podosporaceae, in particular, the relationship between the clades containing *P. anserina* and *P. fimiseda* (clades A and C) is crucial to decide on an optimal naming scheme that minimises taxonomic and practical conflict. Additionally, throughout the phylogeny, there are a number of strains that have been assigned to different species and genera, but likely belong to the same species (e.g. *Thielavia hyalocarpa* and *Zopfiella leucotricha* strain CBS389.84 are identical for ITS and LSU). Additionally, there appears to be undiscovered sexual states of the anamorphic *Cladorrhinum* species, like *C. foecundissimum* with the *A. olerumstrain* CBS120012. Taxonomic re-assignments of these groups should be undertaken; however, without a strong phylogenetic backbone, based on multiple genes and an expanded taxonomic sampling, it seems premature to propose nomenclatural changes.

In recent years, a number of authors have established the use of time-calibrated phylogenies to define ranks from genus up to class for various groups of fungi, although this approach has not been without controversy (Lücking 2019). For the Sordariomycetes, intervals of 150–250 MYA for orders and 50–150 MYA for families have been recommended (Hyde et al. 2017). Both the Sordariales and the Podosporaceae agree well with these values with estimated divergence times of 109.69 MYA and < 76.58 MYA, respectively (Lutzoni et al. 2018), although a comprehensive investigation of divergence amongst the Lasiosphaeriaceae has yet to be conducted. The use of time-calibrated trees to define genera is less common, but a divergence time of ~30 MYA has been suggested previously (Divakar et al. 2017). In this regard, it would be appropriate to define the three clades presented herein as genera as Wang et al. (2019) have done, based on divergence times between *Lasiosphaeria ovina* and *Anopodium ampullaceum* (Hyde et al. 2017), which show similar phylogenetic distances between each other as the three clades of Podosporaceae (Félix 2015). However, time-calibrated phylogenies need not and often should not, set the standard for taxonomic delimitation. One telling example comes from the field of *Fusarium* research. When the ‘one name one fungus’ edict came into effect, it threatened to divide a cosmopolitan group of plant pathogenic fungi that are united in their vegetative morphology and pathophysiology into several genera. To combat this issue, the field pushed for the usage of *Fusarium* for the entire group, despite considerable phylogenetic distance (Geiser et al. 2013). As a result, *Fusarium* encompasses species which diverged more than ~70 MYA (Lutzoni et al. 2018), yet this move ensured the nomenclatural stability of the organisms in question.

When proposing new combinations, one should always ensure to make decisions that will cause the least amount of confusion in literature. In this case, it is clear that, for this goal, the name *P. anserina* should be preserved. Google Scholar returns ~11500 hits to the search query *Podospora anserina*, yet only ~2110 hits for *Triangularia*, the majority of which are due to the use of the word “triangularia” in Latin and have no relation to the genus. There are currently 62967 sequences in Genbank with *Podospora anserina* in the title, while only 76 contain *Triangularia* in the title and lastly, the English Wikipedia page for *Podospora anserina* has had 13897 page views from July 2015 to May 2020, while the English Wikipedia *Triangularia* page has had only 705 views over the same period. The best possible way forward to prevent the re-naming of *P. anserina* or the subsequent instability it will cause in literature is to transfer the type of *Podospora* from *P. fimiseda* to *P. anserina*, despite *P. fimiseda* having been conserved over *Schizothecium fimicola* Corda (Proposal 119). Unfortunately, this process can take many years of debate and the re-assignment of *P. anserina* to *Triangularia* already threatens a peaceful transition. At the very least, if *P. anserina* needs to be assigned to another genus, it should be the type species of that genus in order to prevent further potential nomenclatural changes. Thus, we propose, for the interim, to synonymise *Triangularia* with *Podospora* until a more satisfactory resolution can be made. Once more data are available, it will hopefully be possible to resolve the relationships amongst the three clades. If, in the end, Clade A and B are found to be sisters, then it would require that *Cladorrhinum* sensu Wang et al. (2019) (Clade B here) be synonymised with *Podospora* as well. Alternatively, if Clade A and C are sisters, then *Podospora* could be restricted to these two clades and fewer taxonomic changes would be required. As we ultimately aim to move the type species of *Podospora* to *P. anserina*, we will refrain from making any new combinations at the moment.

In the previous example with *Fusarium*, a divergent group of fungi were classified under one name precisely because the researchers in that field desired unity. In the case of *Podospora*, the only factor necessitating that disparate species fall under one genus is the need to operate within the confines of the ICN. The ultimate goal of the code is to provide taxonomic stability and conformity to the organisms it covers. The nature of studying microscopic fungi has resulted in numerous names with dubious origins and, while obvious fixes are sometimes evident, they are not always possible to enact according to the ICN. It is understandable why the current code is as rigid as it is, but the current editions have seen it become more flexible, which has been advantageous to many fields seeking to solidify tumultuous taxonomy. In the future, we hope that additional data and a permissive code will allow us to enshrine the name *Podospora anserina* indefinitely, settling over a century of nomenclatural friction between taxonomists and other researchers.

## Taxonomy

### Podospora

Taxon classificationFungiSordarialesLasiosphaeriaceae

Ces., Hedwigia 1(15): 103 (1856)

C631C837-89AE-5911-B1A8-7D8F7CB68AFA

4284

#### Type species.

*Podospora fimiseda* (Ces. & De Not.) Niessl, *Hedwigia* 22: 156 (1883).

Syn: *Apiosordaria* Arx & W. Gams, *Nova Hedwigia* 13: 201 (1967).

Syn: *Triangularia* Boedijn, *Annls mycol.* 32(3/4): 302 (1934).

Syn: *Lacunospora* Cailleux, *Cahiers de La Maboké* 6(2): 93 (1969) [1968].

Syn: *Tripterospora* Cain, *Can. J. Bot*. 34: 700 (1956).

Syn: *Philocopra* Speg., *Anal. Soc. cient. argent.* 9(4): (1880).

Syn: *Malinvernia* Rabenh., *Hedwigia* 1: 116 (1857).

Syn: *Pleurage* Fr., *Summa veg. Scand*., Sectio Post. (Stockholm): 418 (1849).

## Supplementary Material

XML Treatment for Podospora

## Supplementary materials

Supplementary material 1 Figure S1 Data type statistical data Explanation note Maximum Likelihood phylogeny of the concatenated analysis of ITS and LSU. Type strains of the focal species are highlighted with coloured boxes. Bootstrap support values are depicted next to their respective branches, but values corresponding to nearly identical sequences are removed for clarity. Branches are proportional to the scale bar (nucleotide substitutions per site). Clades are marked with lateral bars following the topology in Figure 1. https://binary.pensoft.net/file/479674This dataset is made available under the Open Database License (http://opendatacommons.org/licenses/odbl/1.0/). The Open Database License (ODbL) is a license agreement intended to allow users to freely share, modify, and use this Dataset while maintaining this same freedom for others, provided that the original source and author(s) are credited.S. Lorena Ament-Velásquez, Hanna Johannesson, Tatiana Giraud, Robert Debuchy, Sven J. Saupe, Alfons J.M. Debets, Eric Bastiaans, Fabienne Malagnac, Pierre Grognet, Leonardo Peraza-Reyes, Pierre Gladieux, Åsa Kruys, Philippe Silar, Sabine M. Huhndorf, Andrew N. Miller, Aaron A. Vogan

Supplementary material 2 Figure S2 Data type statistical data Explanation note Maximum Likelihood phylogeny of Btub1 and Btub2 separately. The outgroup taxa are not resolved as monophyletic and, hence, the rooting was arbitrarily chosen. Type strains of the focal species are highlighted with coloured boxes. Bootstrap support values are depicted next to their respective branches, but values corresponding to nearly identical sequences are removed for clarity. Branches are proportional to the scale bar (nucleotide substitutions per site). Clades are marked with lateral bars, following the topology in Figure 1. https://binary.pensoft.net/file/479675This dataset is made available under the Open Database License (http://opendatacommons.org/licenses/odbl/1.0/). The Open Database License (ODbL) is a license agreement intended to allow users to freely share, modify, and use this Dataset while maintaining this same freedom for others, provided that the original source and author(s) are credited.S. Lorena Ament-Velásquez, Hanna Johannesson, Tatiana Giraud, Robert Debuchy, Sven J. Saupe, Alfons J.M. Debets, Eric Bastiaans, Fabienne Malagnac, Pierre Grognet, Leonardo Peraza-Reyes, Pierre Gladieux, Åsa Kruys, Philippe Silar, Sabine M. Huhndorf, Andrew N. Miller, Aaron A. Vogan

Supplementary material 3 Figure S3 Data type statistical data Explanation note Maximum Likelihood phylogeny of rpb2. Type strains of the focal species are highlighted with coloured boxes. Bootstrap support values are depicted next to their respective branches, but values corresponding to nearly identical sequences are removed for clarity. Branches are proportional to the scale bar (nucleotide substitutions per site). Clades are marked with lateral bars, following the topology in Figure 1. https://binary.pensoft.net/file/479676This dataset is made available under the Open Database License (http://opendatacommons.org/licenses/odbl/1.0/). The Open Database License (ODbL) is a license agreement intended to allow users to freely share, modify, and use this Dataset while maintaining this same freedom for others, provided that the original source and author(s) are credited.S. Lorena Ament-Velásquez, Hanna Johannesson, Tatiana Giraud, Robert Debuchy, Sven J. Saupe, Alfons J.M. Debets, Eric Bastiaans, Fabienne Malagnac, Pierre Grognet, Leonardo Peraza-Reyes, Pierre Gladieux, Åsa Kruys, Philippe Silar, Sabine M. Huhndorf, Andrew N. Miller, Aaron A. Vogan

Supplementary material 4 Table S1 Data type molecular data Explanation note List of primers used to generate sequence data. https://binary.pensoft.net/file/479677This dataset is made available under the Open Database License (http://opendatacommons.org/licenses/odbl/1.0/). The Open Database License (ODbL) is a license agreement intended to allow users to freely share, modify, and use this Dataset while maintaining this same freedom for others, provided that the original source and author(s) are credited.S. Lorena Ament-Velásquez, Hanna Johannesson, Tatiana Giraud, Robert Debuchy, Sven J. Saupe, Alfons J.M. Debets, Eric Bastiaans, Fabienne Malagnac, Pierre Grognet, Leonardo Peraza-Reyes, Pierre Gladieux, Åsa Kruys, Philippe Silar, Sabine M. Huhndorf, Andrew N. Miller, Aaron A. Vogan

Supplementary material 5 Table S2 Data type statistical data Explanation note Analysed data matrices. https://binary.pensoft.net/file/479678This dataset is made available under the Open Database License (http://opendatacommons.org/licenses/odbl/1.0/). The Open Database License (ODbL) is a license agreement intended to allow users to freely share, modify, and use this Dataset while maintaining this same freedom for others, provided that the original source and author(s) are credited.S. Lorena Ament-Velásquez, Hanna Johannesson, Tatiana Giraud, Robert Debuchy, Sven J. Saupe, Alfons J.M. Debets, Eric Bastiaans, Fabienne Malagnac, Pierre Grognet, Leonardo Peraza-Reyes, Pierre Gladieux, Åsa Kruys, Philippe Silar, Sabine M. Huhndorf, Andrew N. Miller, Aaron A. Vogan
