# Supplementary figures and images for: The taxonomy of the model filamentous fungus Podospora anserina
Source: MycoKeys. 2020 Nov 25;75:51–69. doi: 10.3897/mycokeys.75.55968 (PMC7710671; doi:10.3897/mycokeys.75.55968)

# ITS + LSU

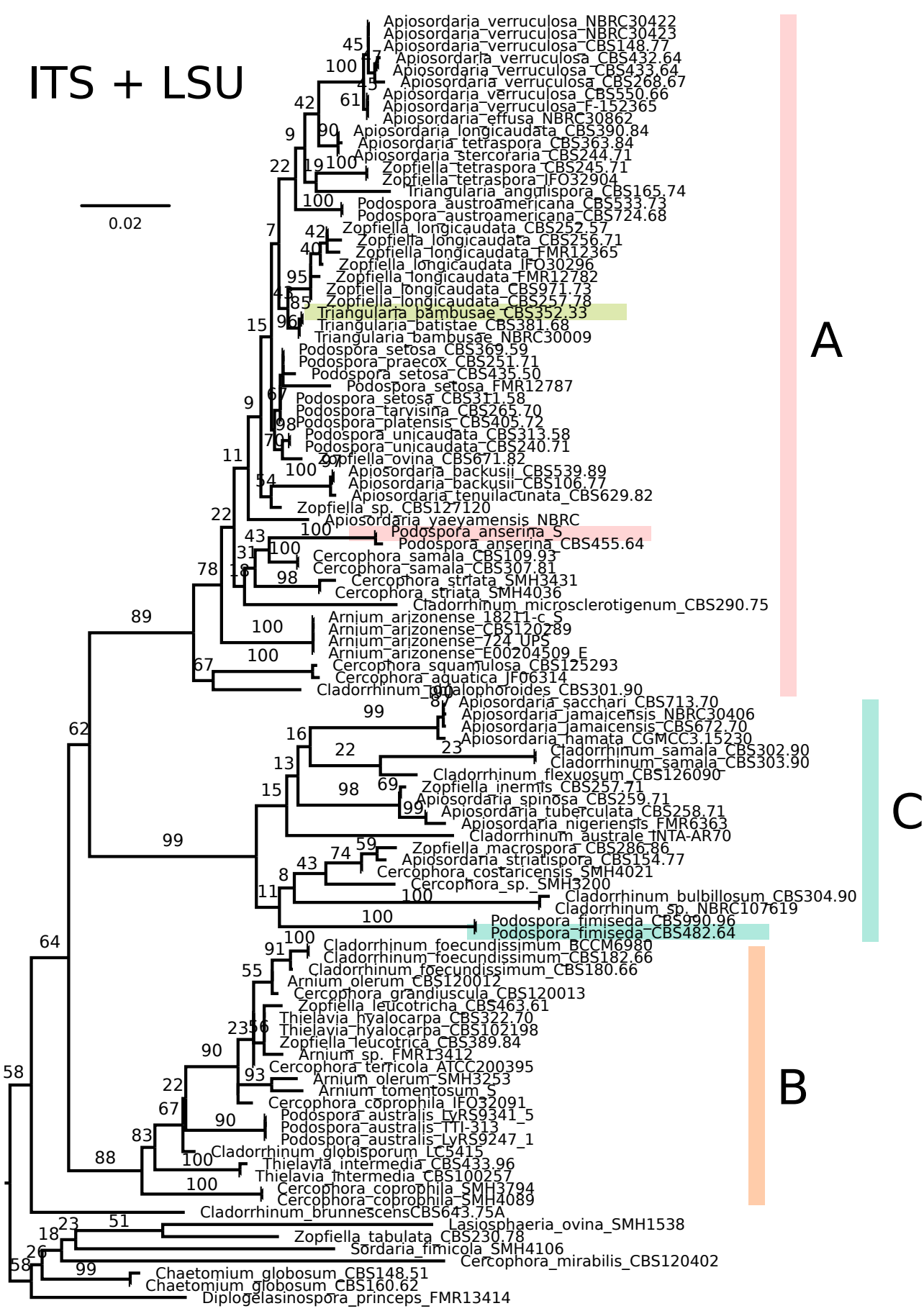

Supplement: Supplementary material 1 — Figure S1 [file mycokeys-75-051-s001.pdf]

# Btub1

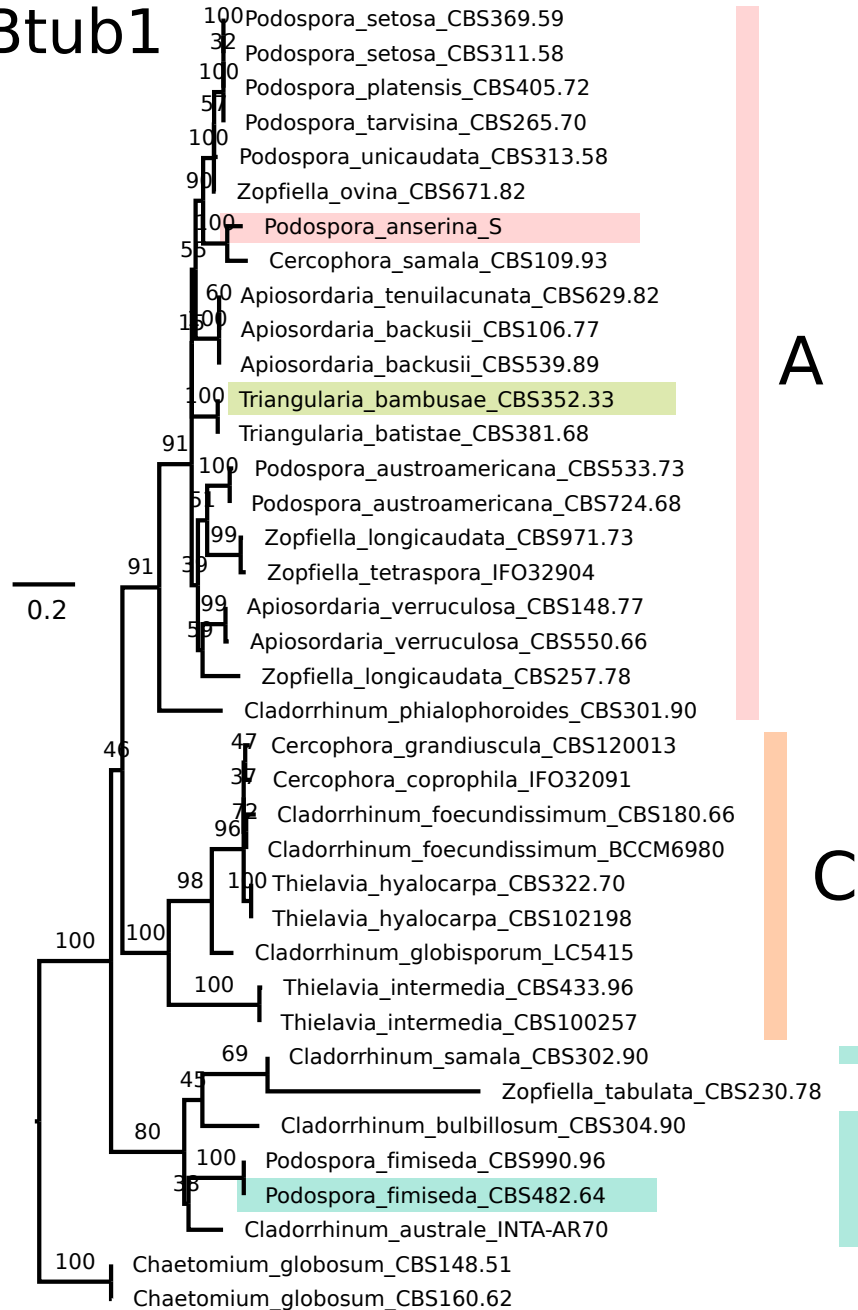

# Btub2

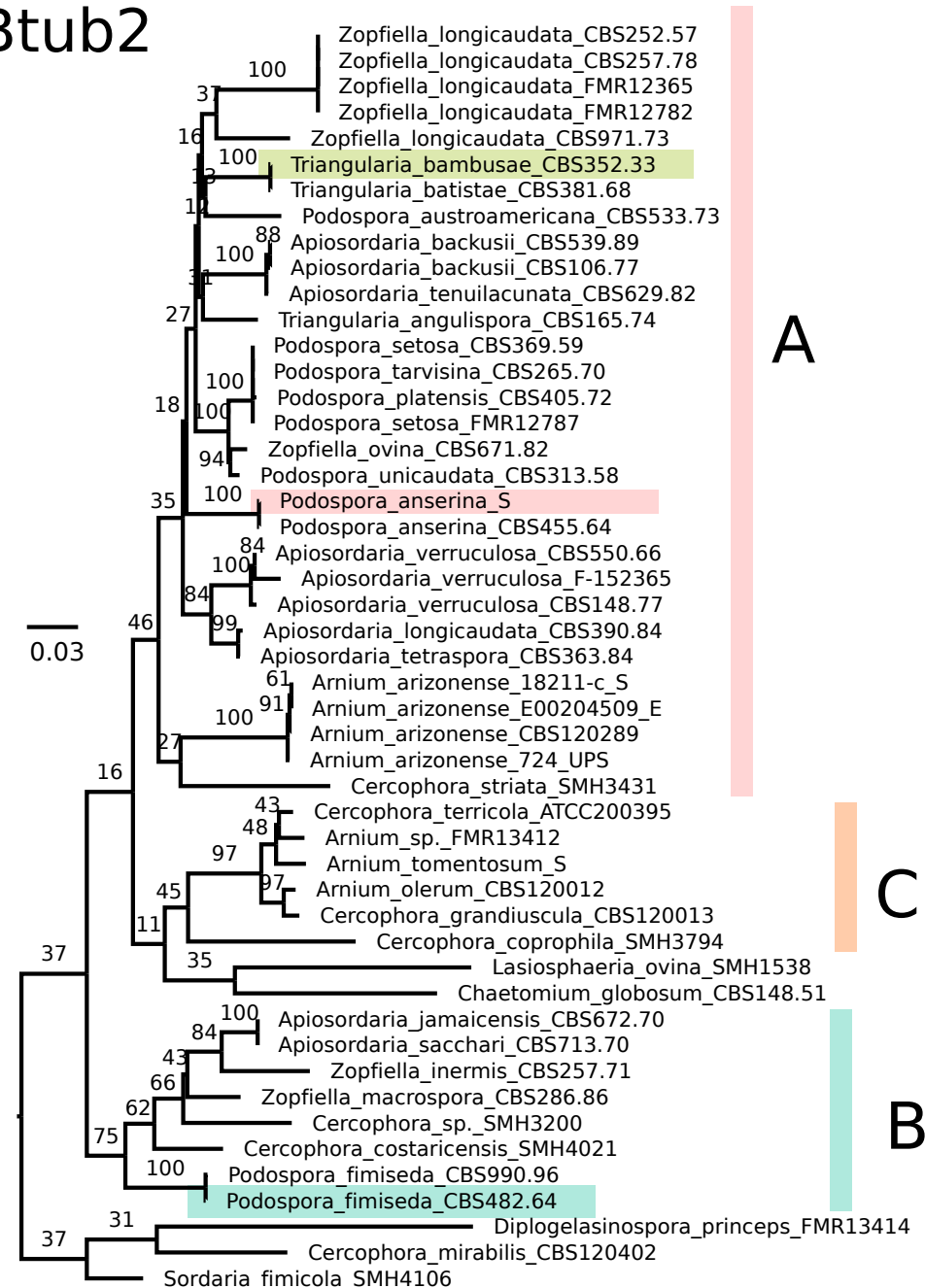

Supplement: Supplementary material 2 — Figure S2 [file mycokeys-75-051-s002.pdf]

rpb2

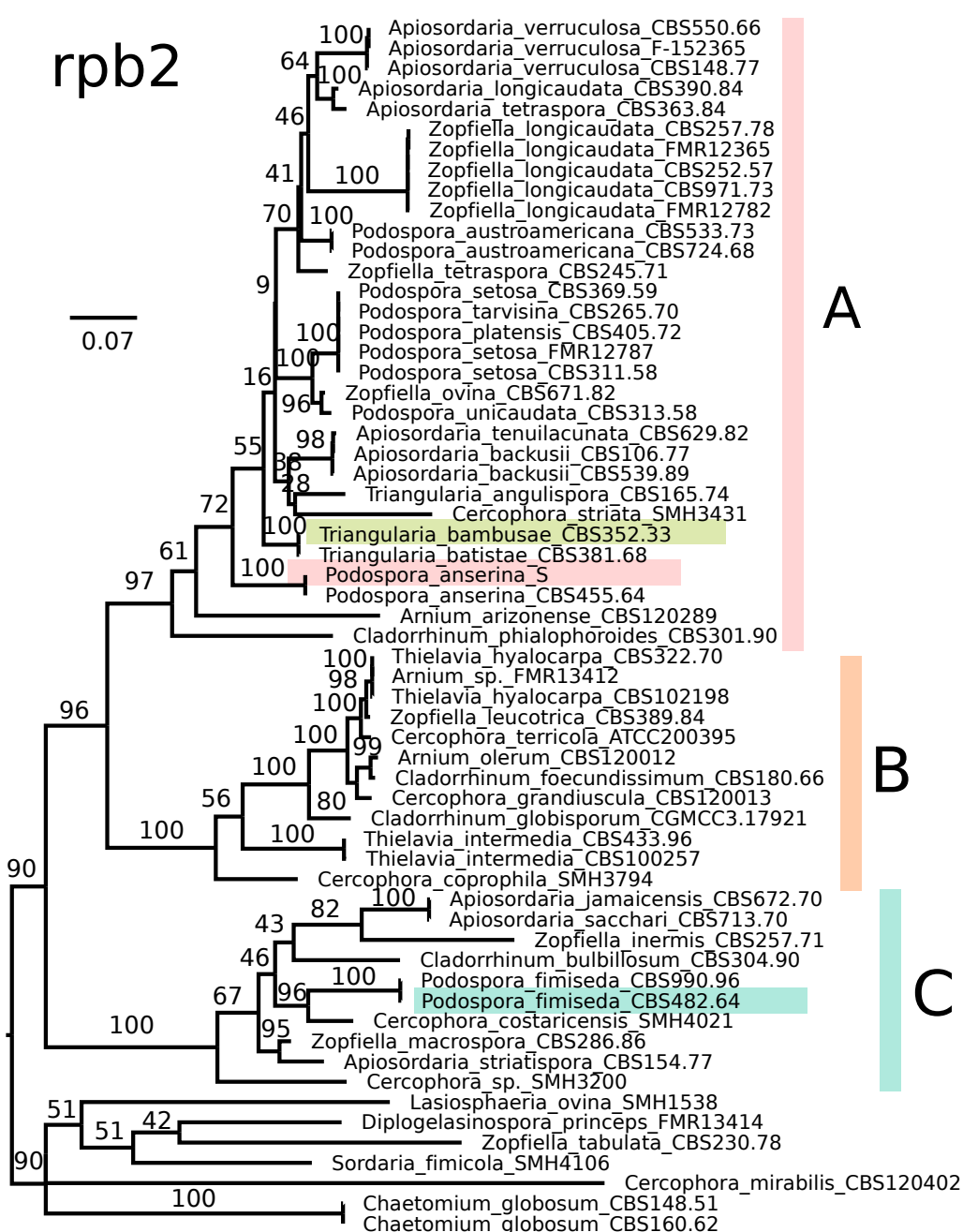

Supplement: Supplementary material 3 — Figure S3 [file mycokeys-75-051-s003.pdf]
